# Cortical Contributions to Attentional Orienting and Response Cancellation in Action Stopping

**DOI:** 10.1111/psyp.70397

**Published:** 2026-09-07

**Authors:** Sarah A. Kemp, Sauro Salomoni, Pierre‐Louis Bazin, Luke Pash, Rebecca J. St George, Mark R. Hinder

**Affiliations:** ^1^ School of Psychological Sciences, College of Health and Medicine University of Tasmania Hobart Australia; ^2^ Department of Psychology University of Amsterdam Amsterdam the Netherlands; ^3^ Wicking Dementia and Research Education Centre University of Tasmania Hobart Australia; ^4^ Full Brain Picture Analytics Leiden the Netherlands

## Abstract

Action cancellation involves the termination of planned or ongoing movement, likely initiated in the cortex by the pre‐supplementary motor area (preSMA) and inferior frontal gyrus (IFG). It is frequently examined using the stop‐signal task; electromyography (EMG) studies have shown action cancellation (in stop trials) can involve the partial activation of the responding muscles, which does not result in an overt behavioral response. The neural correlates of these partial responses are not well understood. Here, we combined functional near‐infrared spectroscopy (fNIRS) and EMG to examine the neural correlates of terminated actions (partial responses) in a response‐ and stimulus‐selective stop signal task, controlling for attentional confounds by comparing neural activity in stop and ignore trials. fNIRS analyses revealed increased preSMA activity in successful stop compared to ignore trials, but no such differences in the IFG, consistent with postulations that the preSMA is involved uniquely in action cancellation, while the IFG responds generically to unexpected stimuli. Critically, preSMA activity was greater in trials without partial muscle activation, potentially due to proactive inhibitory processes pre‐emptively suppressing motor output in these trials. These findings advance understanding of some of the neurophysiological dynamics involved in action cancellation and highlight the utility of combining fNIRS with EMG in this domain.

## Impact Statement

Partial response EMG is near‐ubiquitous in the action cancellation literature and assumed to represent cancellation itself, initiated from the preSMA. We examined cortical activity using fNIRS and, surprisingly, found greater preSMA activity when partial responses were absent, challenging current assumptions and providing the first fNIRS‐based evidence of action cancellation processes.

## 1 Introduction

Action cancellation is the sudden termination of planned or already‐initiated movement in response to unexpected stimuli (Logan 1981). It is a fundamental part of everyday motor control and is impaired in a number of neurological and psychiatric conditions, including Parkinson's disease and attention‐deficit hyperactivity disorder (Coxon et al. 2012; Jahanshahi et al. 2015; Senkowski et al. 2024).

Action cancellation is thought to be mediated by two cortico‐basal ganglia pathways, the hyperdirect and indirect pathways. The indirect pathway likely originates in the pre‐supplementary motor area (preSMA) and involves a broad range of basal ganglia areas, while the hyperdirect pathway originates in the (right) inferior frontal gyrus (IFG), and includes only the subthalamic nucleus (STN) and the globus pallidus interna (Bingham et al. 2023; Coudé et al. 2018; Nambu et al. 2002). The “pause‐then‐cancel” model postulates that action cancellation occurs in two stages, underpinned by these two pathways (Diesburg and Wessel 2021; Schmidt and Berke 2017). When an unexpected stimulus is presented, the IFG sends a rapid “pause” signal via the hyperdirect pathway, increasing inhibition to all muscles and delaying the movement without modifying it. The slower “cancel” process, initiated in the preSMA, then modifies the movement according to contextual requirements, via the indirect pathway (Diesburg and Wessel 2021; Tatz et al. 2021).

The standard paradigm to assess action cancellation is the stop‐signal task (SST), where participants make a predetermined motor response in each trial (e.g., a button press) in response to a “go” signal. On a subset of trials (generally 25%–33%), a stop signal appears shortly after the go signal, requiring that the participant try to cancel their response (Verbruggen et al. 2019). However, a longstanding concern of the SST has been that the infrequent stop signal engages attentional orienting mechanisms in addition to action cancellation, confounding attempts to identify neurobiological correlates specific to action cancellation (Sharp et al. 2010). Thus, *stimulus‐selective SSTs* incorporate additional infrequent “ignore” stimuli, which require attentional orienting but do not necessitate action cancellation (e.g., Boehler et al. 2010; Chatham et al. 2012; Weber et al. 2023). Additionally, while most SSTs require the entire action to be cancelled in a stop trial, *response‐selective SSTs* can be used to examine “selective” stopping, where only part of the ongoing response is cancelled (Weber et al. 2026). This is thought to more closely resemble real‐world scenarios, where we are more likely to adjust ongoing movements rather than freeze entirely when something unexpected occurs. Response‐selective stopping is associated with a response delay in the continuing effector, referred to as the *stopping delay* or *stopping interference effect* (Gronau et al. 2024; Wadsley et al. 2022). This may arise from an initial, effector‐wide inhibition (triggered by the “pause” process) in response to the stop signal; however, the underlying neurophysiological mechanisms remain incompletely characterized.

Electromyography (EMG) is ideal for capturing the electrophysiological changes that occur during movement execution and action cancellation (Raez et al. 2006). It can be used to detect *partial responses*: instances where EMG amplitude increases above a certain threshold without resulting in an overt behavioral response (also referred to as *prEMG* or *partial bursts*; Raud et al. 2022; Salomoni et al. 2025; Thunberg et al. 2024). These are thought to capture instances where an initiated action is terminated at the muscle before producing a behavioral response (Atsma et al. 2018; Raud et al. 2022). Partial responses reflect dynamic changes that can be measured with high temporal sensitivity, allowing distinctions to be made between trials with no behavioral output (MacDonald et al. 2017; Wadsley and Greenhouse 2024). However, the neural mechanisms underlying these dynamics remain unclear, particularly regarding if neural signatures differ between trials with and without partial responses.

Functional near‐infrared spectroscopy (fNIRS) is a non‐invasive, optical neuroimaging technique that estimates concentration changes in oxygenated (Hb) and deoxygenated hemoglobin (dHb; Pinti et al. 2020). Changes to Hb and dHb concentration are associated with neural activity, similar to the blood‐oxygenation level‐dependent (BOLD) signal fundamental to functional magnetic resonance imaging (fMRI; Heeger and Ress 2002). fNIRS is a portable and convenient neuroimaging technique and is well‐suited for imaging brain regions relatively close to the skull, such as the preSMA and IFG (Lu et al. 2025; Su et al. 2023). It has previously been used for examining changes in neural activity induced by the stop signal in both clinical and healthy populations (Afonso Junior et al. 2023, 2025; Healey et al. 2024).

In the present study, we combined fNIRS and EMG within a response‐selective and stimulus‐selective SST to characterize cortical and muscular dynamics associated with complex action cancellation. By contrasting trials with stop and ignore signals, we aimed to dissociate the neural activity related to attentional capture from that specifically associated with action cancellation. Additionally, we examined if trials with interrupted motor output (partial EMG responses) were associated with distinct neural signatures compared to those without them.

## 2 Methods

### 2.1 Participants

Thirty participants (mean age = 28.33 years, SD = 7.33, all right‐handed, 19 females) were recruited via the University of Tasmania's psychology research participation system or personal invitation. All participants had normal or corrected‐to‐normal vision, no color blindness, and no history of neurological or major psychiatric disorders (including ADHD) and were self‐reported right‐handed. As compensation for their time, participants received either 2 h of research credit or a $20 AUD shopping voucher. All participants gave written informed consent. One participant exhibited a proportion of successful stop trials (78.67%) that was over the recommended range (Verbruggen et al. 2019) and was thus excluded from the analysis. The fNIRS data of another participant was not usable due to a technical error. The final sample size was thus 29 for the behavioral and EMG components of the analysis, and 28 for the fNIRS component. The study was approved by the Tasmanian Human Research Ethics Committee (#H0014865) and conducted in accordance with the Declaration of Helsinki.

### 2.2 Stop‐Signal Task (SST)

**FIGURE 1:**
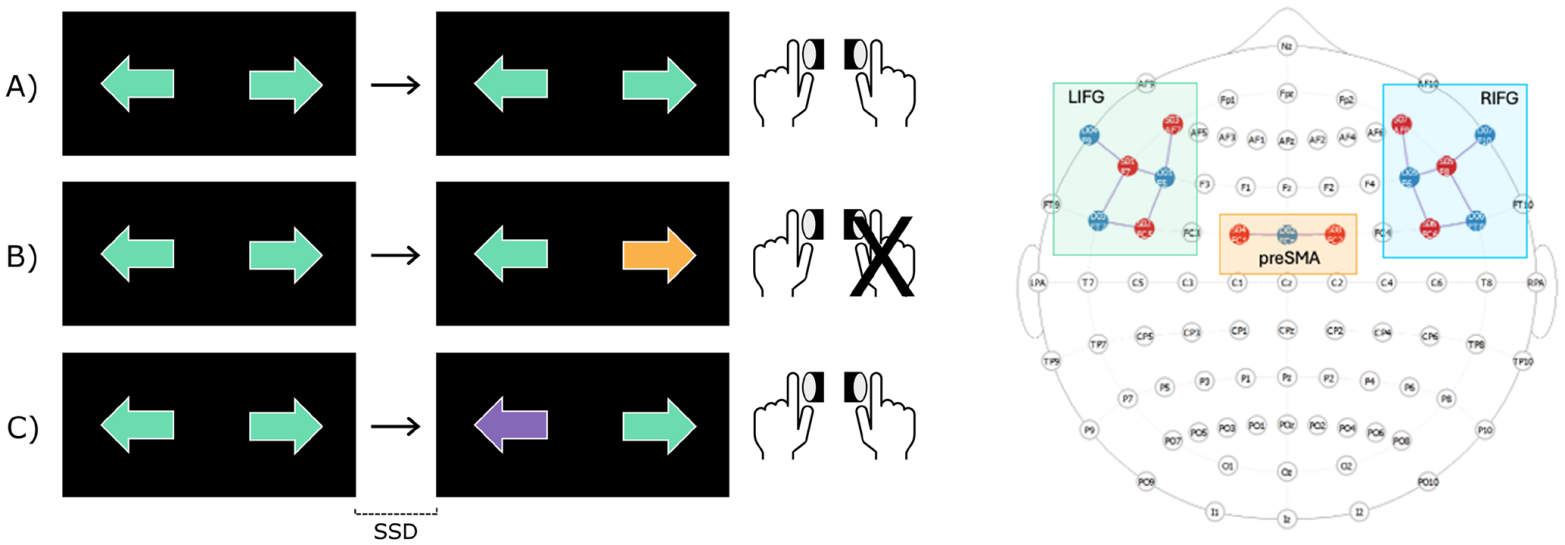
Schematic of the experimental task (left) and the custom montage used in the fNIRS set up (right). *Task*: Trials could be either go trials, stop trials, or ignore trials. Each trial started with the presentation of two green arrows, to which participants made fast‐as‐possible bimanual responses. In a go trial (A), the arrows remained green throughout the trial. In a stop trial (B), one of the arrows turned orange, indicating participants should try to cancel that component of the response and give a unimanual response. In an ignore trial (C), one of the arrows turned purple. Participants were to ignore this color change and continue the bimanual response. The stop‐signal delay (SSD) determined the onset of the color change in each stop and ignore trial. Note that either arrow could change color in a given trial, but only a right‐sided stop trial (B) and a left‐sided ignore trial (C) are illustrated here. *fNIRS*: Custom montage used to capture changes in hemoglobin concentration in the left and right inferior frontal gyri (LIFG and RIFG; indicated in green and blue, respectively) and pre‐supplementary motor area (preSMA; indicated in orange). The montage comprised 14 channels (8 sources, 7 detector). Six channels were used for the LIFG, six for the RIFG, and two for the preSMA.

A response‐ and stimulus‐selective SST was used, with go trials, ignore trials, and stop trials. Each trial began with a fixation cross, the duration of which was randomly drawn from a truncated exponential distribution (range: 500–2500 ms, median duration ≈ 1130 ms). This randomization reduced the predictability of the go signal onset, ensuring participants were responding to the go signals themselves and not responding prematurely in anticipation (Verbruggen et al. 2019). The go signal was two green arrows. When they appeared, participants would make a bimanual, inwards button press (simultaneously with the left and right index finger) against vertically mounted buttons as quickly as possible (see Figure 1). The response window was 2000 ms.

In a stop trial, one of the green arrows would turn orange at a precise delay time after the go onset, indicating that the participant should withhold their response with that hand but still execute a button press with the other hand (response‐selective inhibition). In an ignore trial, one of the arrows would turn purple after a delay. Here, participants were required to ignore the color change, and make the standard bimanual response (stimulus‐selective inhibition). Feedback on trial success and response time was then presented for 2000 ms. The latency between the onset of the go and stop signals (stop signal delay; SSD) was modified after each trial using a staircase procedure (Verbruggen et al. 2019). If a participant stopped successfully in a stop trial (i.e., gave the correct unimanual response), the SSD was increased by 50 ms on the next trial. Conversely, if a participant failed to stop the bimanual response or gave the wrong response (i.e., stopped the wrong hand), the SSD was decreased by 50 ms, making it easier for the participant to inhibit their response in the next stop trial. This iterative adaptation ensured that each participant was able to appropriately inhibit their response on approximately 50% of trials (Verbruggen et al. 2019). SSDs were tracked independently for each hand. The onset of the ignore signal was determined by the current SSD for that hand.

### 2.3 Procedure

Participants were seated approximately 80 cm from a computer monitor, with their forearms pronated and arms resting on a desk, shoulder width apart. Each index finger rested against a custom‐made response button. The buttons were mounted in the vertical plane, such that pressing a button required participants to abduct their index fingers (Figure 1). This maximized activation of the first dorsal interrosei (FDI) muscles, as they act as agonists in this action (this also improves the signal‐to‐noise ratio [SNR] and the detection of EMG bursts; see 2.3.1 “Electromyography”). Button presses were registered via the Black Box Toolkit USB response pad and recorded using PsychoPy3 and Signal4 (Cambridge Electronic Design Ltd.; Peirce et al. 2019). The SST was run via custom‐written code in PsychoPy3 (Peirce et al. 2019).

After receiving instructions, participants completed 20 practice trials, which comprised 10 go trials, four ignore trials (two ignore left, two ignore right), and six stop trials (three stop left, three stop right). Following the practice, participants performed a total of 680 trials divided in 10 blocks (68 per block). Of these 680 trials, 450 were go trials (66%), 150 were stop trials (22%; 75 left and 75 right), and 80 were ignore trials (12%; 40 left, 40 right). Each 68‐trial block contained the same proportions of trials, that is, 45 go trials, 15 stop trials, and 8 ignore trials. After each block, participants had an opportunity to rest before continuing. The first three participants completed more trials than the rest of the cohort, completing approximately 90 trials per block, that is, 900 trials in total. However, it was found that the overall length of the experiment setup was longer than had been estimated during piloting, and trial numbers were thus reduced to minimize any possible fatigue effects. After careful inspection of the dataset, it was determined that these six participants did not differ from the remainder of the cohort in terms of their performance on stop and ignore trials, and had a similar reaction time (RT) distribution, indicating they performed similarly despite the longer experiment. All data from these participants were therefore retained in the analysis to maintain statistical power.

In addition to feedback at the end of each trial, participants received feedback at the end of each block, telling them their mean RT for that block. If the mean RT was more than 100 ms slower than in the previous block, additional feedback was provided, reminding them to respond as quickly as possible. This was to minimize proactive slowing strategies, as these create problems in the analysis of stop‐signal data (Verbruggen et al. 2019; Verbruggen and Logan 2009) and are associated with distinct neural correlates compared to fast‐as‐possible responses, especially in inferior frontal regions (Messel et al. 2019; Sánchez‐Carmona et al. 2016).

#### 2.3.1 Electromyography (EMG)

EMG was recorded using disposable adhesive electrodes (Ag/AgCl). Two electrodes were positioned in a belly‐tendon montage over the FDI of each hand, with a ground electrode placed on the head of the ulna. Recordings were synchronized via a TTL pulse from the PsychoPy software and made using the software Signal4 (Cambridge Electronic Design Ltd). The analog EMG signals were bandpass filtered at 20–1000 Hz, amplified 1000 times, sampled at 2000 Hz (CED Power 1401 and CED 1902, Cambridge, UK), and saved into a PC for offline analysis.

#### 2.3.2 Functional Near‐Infrared Spectroscopy (fNIRS)

Near‐infrared light intensity changes in the left IFG (LIFG), right IFG (RIFG), and preSMA were recorded with a custom fNIRS montage. Recordings were made with NirStar v15.3 with a sampling rate of approximately 7.82 Hz. Synchronization was achieved using a digital TTL signal from PsychoPy. The system (NirSport, NIRs Medizintechnik GmbH, Berlin) emits near‐infrared light at 760–850 nm wavelengths to capture haemodynamic changes in the cortical tissue up to 1.5 cm deep (Brigadoi and Cooper 2015; Pinti et al. 2020). The locations of the regions of interest (ROIs) were determined using masks from the Automated Anatomical Labelling 2 (AAL2) atlas. The AAL2 provides parcellation of the orbitofrontal cortex based on the Montreal Neurological Institute (MNI) standardized and spatially‐normalized brain atlas (Rolls et al. 2015; Tzourio‐Mazoyer et al. 2002).

The montage was determined using the fNIRS Optodes' Location Decider (fOLD) toolbox v2.2 (Zimeo Morais et al. 2018) and comprised 14 channels, with eight sources and seven detectors (see Figure 1). Six channels were used to capture the LIFG, six for the RIFG, and two for the preSMA. Given the small number of channels used to capture the preSMA, the limited spatial resolution of fNIRS, and the proximity of the preSMA to the midline (Hiroshima et al. 2014), this region was not split into right and left hemisphere. The channels and their approximate coordinates in MNI space (estimated using fOLD) are given in Table 1.

**TABLE 1:** The custom fNIRS montage with the approximate MNI coordinates for each channel. Montage was determined using the fNIRS Optodes' Location Decider (fOLD) toolbox (Zimeo Morais et al. 2018).

Region	Channels		MNI coordinates (mm)		
	Source	Detector	*x*	*y*	*z*
Left IFG	FC5	FT7	−59	11	9
	FC5	F5	−56	24	20
	F7	F5	−53	37	6
	F7	FT7	−54	21	−4
	F7	F9	−43	31	−12
	AF7	F5	−47	46	6
Right IFG	FC6	FT8	61	11	8
	FC6	F6	58	24	18
	F8	FT8	57	21	−4
	F8	F6	55	36	5
	F8	F10	52	30	−13
	AF8	F6	48	46	5
(pre)SMA	FC1	FCz	−13	12	67
	FC2	FCz	14	13	66

### 2.4 Data Preprocessing

#### 2.4.1 EMG Data

**FIGURE 2:**
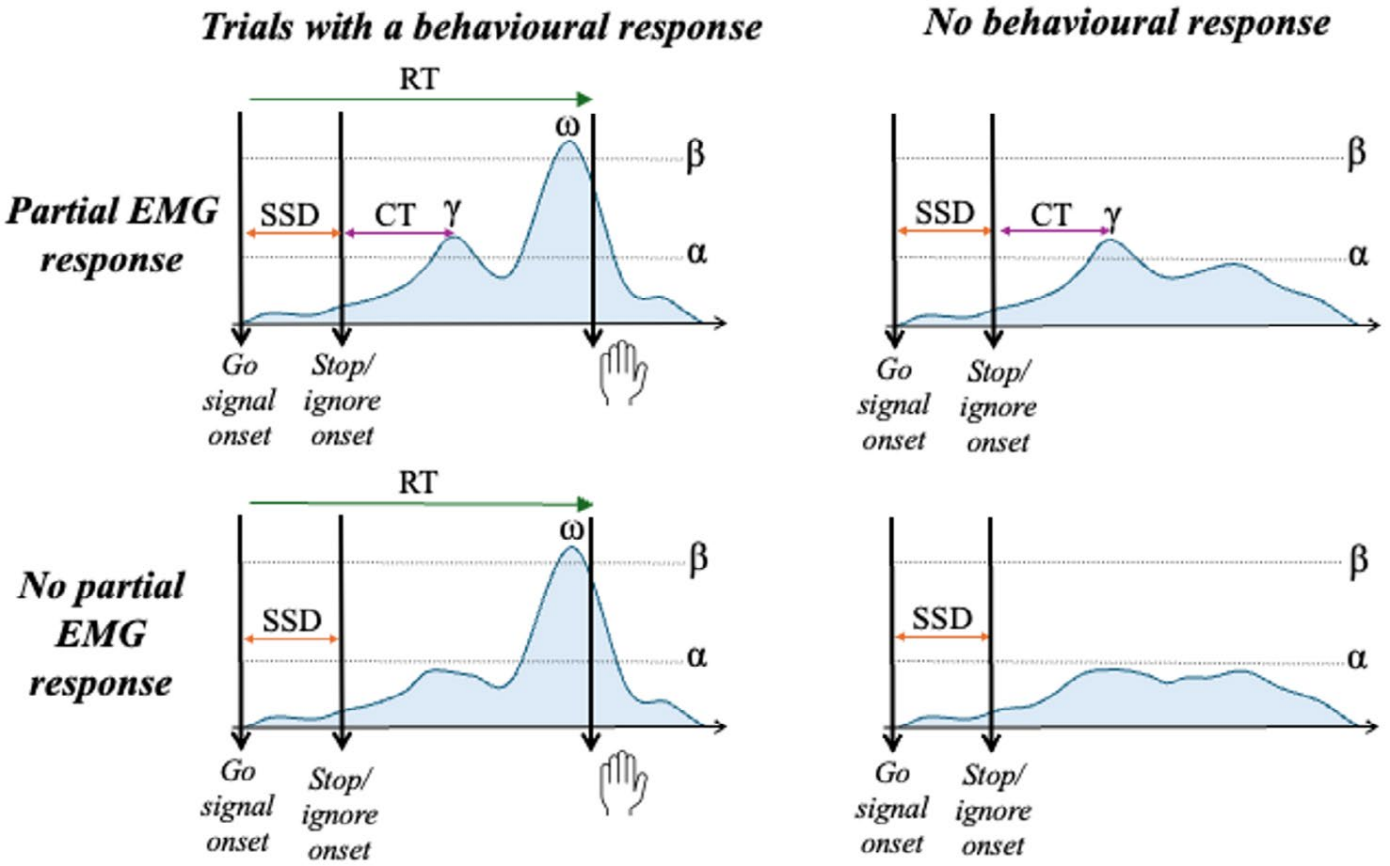
An illustrative schematic of EMG traces after presentation of a stop or ignore signal, showing EMG measures of interest. The onset of the stop or ignore signal is determined by the stop‐signal delay (SSD). The reaction time (RT) is the time between onset of the go signal and the button press. Partial responses are instances where the EMG signal crosses the threshold *α* without having a subsequent button press. Response‐generating EMG bursts are the increase in EMG closest to the recorded button press that cross the threshold *β*. Measures of interest include cancel time (CT; the latency between stop/ignore signal onset and the peak of the partial response *γ*), RT, and the amplitude of the response‐generating EMG burst (*ω*).

EMG preprocessing was performed using custom scripts, implemented in MATLAB R2017B (MathWorks 2017). The signals were filtered using a fourth‐order bandpass Butterworth filter at 20–500 Hz. To detect the precise onset and offset times of the EMG responses, we used a single‐threshold algorithm as per Hodges and Bui (1996). A sliding window of 500 ms was used to find the segment in the timeseries with the lowest root mean squared amplitude, which was then used as a baseline to estimate the latencies of EMG responses. Signals from each trial were rectified and smoothed by lowpass filtering at 50 Hz to obtain EMG envelopes, which were used to estimate the amplitude of each burst. EMG points of interest are depicted in Figure 2.

EMG responses were categorized as instances where the amplitude of the smoothed signal exceeded a threshold of three standard deviations (SD) above baseline (*α* in Figure 2). The responses were considered either *response‐generating*, where this signal increase preceded an overt button press, or *partial responses*, where the signal crossed the threshold but did not result in a button press. Onset and offset times were also considered in the identification of these responses. Response‐generating EMG bursts were defined as the last burst prior to the recorded button press (indicated by *ω* in Figure 2). Similar to our previous studies (Salomoni et al. 2023; Weber, Salomoni, St George, et al. 2024), partial EMG responses were identified as the first instance of the signal crossing the threshold in a trial (*γ* in Figure 2), as long as this increase occurred after the SSD and the amplitude of this response was greater than 10% of the average peak of the successful go trials for that participant. Partial response onset had to occur before any subsequent response‐generating bursts in that trial; any partial movements close to the button press (simultaneous with a response‐generating burst in the responding hand) are likely attributable to mirror activity or, if occurring after the press, due to other activation unrelated to the task. EMG responses separated by less than 20 ms were merged together, as these are unlikely to represent distinct activations. For each response detected (response‐generating and partial responses), we extracted the times of the onset, peak, and offset. The latency of the peaks for partial responses and response‐generating bursts, as well as the amplitude of the response‐generating bursts, were used as measures of interest in the analysis (see Figure 2 and 2.5.2 “EMG analysis”).

#### 2.4.2 fNIRS Data

fNIRS data were processed using Homer3 v1.80.2, implemented in MATLAB R2017b (Huppert et al. 2009; MathWorks 2017). In the processing stream, missing values were replaced by spline interpolation of the existing values using the *PreprocessIntensity*_NAN Homer3 function. *PruneChannels* was then used to remove channels with an SNR below 2. Of the 14 channels in the montage, six participants had one noisy channel (resulting in the removal of 7% of their data), two participants had two noisy channels (14% of their data removed), and one had three (21% removed). The data were then converted from light intensity to optical density using the *Intensity2OD* Homer function, and motion artifacts were removed using *MotionCorrectPCArecurse*. High‐ and low‐pass filtering (0.005 and 0.5 Hz, respectively) was applied using a third‐order bandpass Butterworth filter via *BandpassFilt*. The optical density data were then converted to concentrations of Hb and dHb by the modified Beer–Lambert law using *OD2Conc*. A configuration file showing all functions and values for the processing stream can be found at https://osf.io/ztb5m/.

**FIGURE 3:**
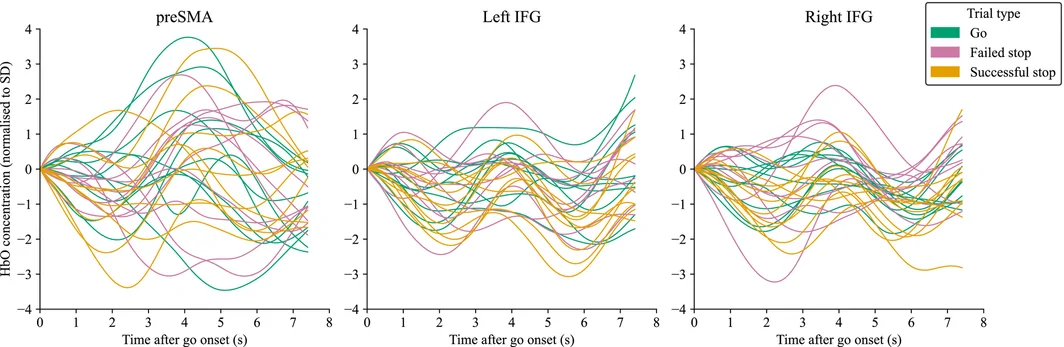
Sample trials from a representative participant, showing changes in Hb concentration in the preSMA and left and right IFG in the 8 s following go signal onset (time 0). Changes in neural activity triggered by the stimulus are reflected in Hb concentration changes, typically appearing with a 3–6 s delay. For analysis, the peak Hb concentration (local maximum) in the 2–7 s following go signal presentation was extracted per trial.

Increases in neural activity create a subsequent increase in the haemodynamic response, influencing both Hb and dHb concentration (Heeger and Ress 2002; Watanabe et al. 2017). Data analyses focused on Hb concentration, as this measure has a higher SNR than dHb and is most correlated with the fMRI BOLD response (Eggebrecht et al. 2012; St George et al. 2021, 2022). For each participant, the channels corresponding to each region (preSMA, RIFG, LIFG) were averaged to obtain a single timeseries per ROI. Trial‐level timeseries were then extracted over a 10‐s time window for each trial, starting 500 ms before go onset, resulting in a waveform of haemodynamic (Hb concentration) change for each trial per ROI. Figure 3 shows an example of the waveforms of a single participant, with Hb concentration changes in the three ROIs for a sample of go, failed stop, and successful stop trials.

To assess differences in neural activity between different trial types, standard fNIRS approaches generally involve averaging the waveforms across trials, from which summary values (e.g., peak or mean Hb concentration) can be extracted per condition and participant (Healey et al. 2024; St George et al. 2021). However, such averaging discards trial‐by‐trial variability, implicitly treating the participant‐level estimates as precise, which is an assumption unlikely to hold in practice. There are multiple sources of measurement error (e.g., signal quality fluctuations, movement artifacts, electrophysiological noise), which can vary across trials and conditions, increasing measurement noise, and there may also be systematic differences between conditions. For instance, stop trials are fewer in number and more cognitively demanding than go trials, meaning they likely are associated with greater within‐condition variability (for examinations of the neural signatures of stop trials across different contexts, see Boehler et al. 2010, 2011; Claffey et al. 2010; Dykstra et al. 2020; Lavallee et al. 2014). This heterogeneity biases group‐level estimates, particularly when there are unequal trial numbers (Gelman and Hill 2006). Here, rather than averaging across trials, we extracted peak Hb concentration from each trial individually, enabling the application of hierarchical (mixed‐effects) models, which account for within‐ and between‐subject variability simultaneously (Boisgontier and Cheval 2016; Gelman and Hill 2006; Hesselmann 2018; for further discussion on the application of hierarchical models to neuroimaging data, see Aarts et al. 2014; Fiedler 2011; Yu et al. 2022).

For each participant, we first removed outliers by removing any trials whose maximum absolute value exceeded 3 × 𝐼𝑄𝑅 (interquartile range) of maximum values in that trial type (stop, go, ignore). We then normalized each trial by dividing it by the mean SD of all trials for that participant per ROI. After normalization, the local maximum of Hb concentration in each Trial 2–7 s after the event trigger was extracted using custom Python code. This window was chosen given that changes in Hb concentration relevant to the presented stimulus tend to occur 3–6 s after stimulus presentation (Healey et al. 2024; Pinti et al. 2020). Analysis code for outlier removal, normalizing, and the extraction of trialwise datapoints is shared at https://osf.io/ztb5m/.

### 2.5 Data Analysis

Trials are categorized as either *successful go* trials (two green arrows presented and the participant responds correctly), *failed go* trials (two green arrows presented and the participant makes some response other than a bimanual response), *failed stop* trials (a stop signal is presented but the participant responds with both hands, no hands, or with the wrong hand), *successful stop* trials (a stop signal is presented and the participant gives the correct, unimanual response), *successful ignore* trials (an ignore signal is presented and the participant gives a bimanual response), or *failed ignore* trials (an ignore signal is presented and the participant gives some response other than a bimanual button press). Trials in which the participant responded < 100 ms after go signal onset or slower than the stimulus duration of 2000 ms were excluded from the analysis (0.07% of all trials). Failed go and failed ignore trials were not included in the analyses due to their infrequency (participants responded correctly in 99.92% of go trials and 98.74% of ignore trials). Thus, unless otherwise specified, the terms “go trial” and “ignore trial” refer only to *correct* go and *correct* ignore trials, respectively.

For statistical analysis of the behavioral and EMG data, we used generalized linear mixed models (GLMMs), with either Gaussian distributions and log link functions, or gamma distributions with identity functions. Both approaches are effective in combating the skewness inherent in RT data (Lo and Andrews 2015; Morís Fernández and Vadillo 2020; van der Linden 2006). The fNIRS data contained both positive and negative numbers and was less skewed (i.e., closer to a normal distribution), thus we used standard linear mixed models (LMMs) to assess them, which assume that the data are normally distributed.

In each case, multiple candidate models with varying random effects structures were fit (e.g., random intercepts only vs. random intercepts plus random slopes). Candidate models were compared using Bayesian Information Criterion (BIC; Schwarz 1978). The model with the lowest BIC was selected as the best‐fitting model. All candidate models and their relative fits (BICs) can be found in the Supporting Information. Models were implemented using the *GAMLj* package in jamovi v2.3 (Gallucci 2019; Love et al. 2022). The jamovi output for all analyses, including all candidate models, can be found at https://osf.io/ztb5m/.

#### 2.5.1 Behavioral Analysis

For trials with bimanual responses, RT was quantified as the mean of the left and right button presses, given the close temporal coupling typical between hands (mean participant‐level RT difference between left‐ and right‐handed presses was *M* = 2.8 ms, SD = 10.2). In trials with a unimanual response (successful stop trials), RT was the timing of the single button press. Note that a unimanual button press was also possible in incorrect go and failed ignore trials, but these rarely occurred (0.05% and 0.15% of all trials, respectively) and were not included in the analysis.

In standard SSTs, there is no behavioral measure in successful stop trials as the participant, by definition, has cancelled their response. The stop‐signal reaction time (SSRT) is often used as an estimation of the latent stopping process (Logan and Cowan 1984; Matzke et al. 2013; Verbruggen et al. 2019). In the current paradigm, the selective stopping means that there is an overt behavioral response in every trial, even in a successful stop trial (where there will be a unimanual button press from the unstopped hand). We thus assessed RT using a GLMM with trial type (go, failed stop, successful stop, and ignore) as a factor.

#### 2.5.2 EMG Analysis

For the EMG analysis, we were primarily interested in examining partial responses. We thus focused on successful stop and successful ignore trials, assessing the effects of partial responses on other measures (i.e., RT and fNIRS measures). Partial responses could appear in one or both hands within a trial, so the RT and EMG measures were taken for each hand per trial, rather than being averaged across hands. We assessed the EMG measures in four different ways, as described below.

##### 2.5.2.1 Reaction Time (RT)

We assessed RT differences in trials with and without partial responses using a GLMM with trial type (successful stop, successful ignore) and partial response (present, absent) as factors. For this analysis, we took the RT of the uncued or unstopped hand in each trial. For a stop trial, we took the RT of the continuing hand, that is, the one that wasn't cued to stop. For an ignore trial, we took the RT of the hand for which there was no ignore signal. This was to ensure RT was comparable between stop and ignore trials.

##### 2.5.2.2 Cancel Time (CT)

CT is the latency of the peak amplitude of the EMG signal in a partial response, relative to that trial's SSD (see Figure 2). Previous work has used this measure as a trial‐wise estimate of stopping latency (Jana et al. 2020; Weber et al. 2023) and it is considered a close electrophysiological correlate to SSRT (Raud et al. 2022; Salomoni et al. 2025). We assessed each hand individually per trial, as partial responses could occur in one or both hands in any trial. In this context, hands were classified as cued or uncued, depending on whether the stop or ignore signal appeared on that side. We assessed CT latency using a GLMM with trial type (stop, ignore) and hand (cued, uncued) as factors.

##### 2.5.2.3 Amplitude

We then assessed the amplitudes of response‐ generating bursts in successful stop and ignore trials (given by ω in Figure 2) with and without partial responses. As in the assessment of RT as a function of partial response presence (see above), we here took only the amplitude of the continuing hand, that is, the left hand in a right ignore/stop trial, and the right hand in a left ignore/stop trial. We assessed amplitude using a GLMM with trial type (stop, ignore) and partial response (present, absent) as factors.

##### 2.5.2.4 Action Reprogramming

Finally, we examined the speed of action reprogramming in stop and ignore trials with partial responses by assessing the latency between the peak of the partial response and the peak of the response‐generating EMG burst (ω and γ in Figure 2, respectively). Again, in order to make the stop and ignore trials more comparative, we took the continuing (non‐cued) hand (e.g., the left hand in a right ignore trial). We assessed these latencies using a GLMM with trial type (stop, ignore) as a factor.

#### 2.5.3 fNIRS Analysis

As with the EMG analysis, we focused our analyses on successful stop and ignore trials, that is, trials that could have partial responses. For each of the three ROIs, we assessed Hb concentration in stop and ignore trials with trial type (stop, ignore) and partial response (present, absent) as factors using an LMM.

#### 2.5.4 Correlations

Finally, we conducted exploratory correlation analyses examining the relationship between mean fNIRS peak Hb concentration per trial type and corresponding behavioral and EMG indices. For each participant and ROI, mean peak Hb concentration in go, ignore, failed stop, and successful stop trials was correlated with mean go RT, mean ignore RT, mean failed stop RT, and mean CT respectively, yielding 12 Pearson correlations in total. *p*‐values were corrected for multiple comparisons using the Benjamini‐Hochberg false discovery rate (FDR: Benjamini and Hochberg 1995) procedure.

## 3 Results

### 3.1 Behavioral Results

**FIGURE 4:**
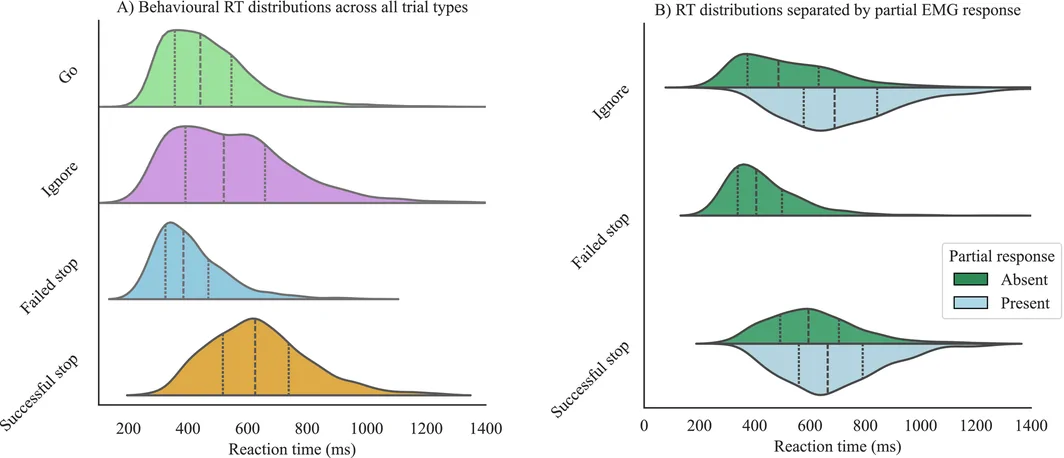
Reaction time (RT) distributions with quartiles for (A) all trial types and (B) stop and ignore trials, split by the presence of partial responses. Dotted lines represent the 25th and 75th percentiles; dashed lines represent the 50th. Panel (A) shows RT distributions for all four trial types (go trials, ignore trials, failed stops, and successful stops). There were significant differences between RT in all four trial types (all 𝑝 < 0.001; see 3.1). (B) Shows the RT distributions for successful stop and ignore trials, split by the presence of partial EMG responses. All RT differences were significant (all 𝑝 < 0.001; see 3.2.1). Note that failed stop trials were not included in the analysis for 3.2 as they do not contain partial responses, but are depicted here for comparative purposes.

Descriptive statistics summarizing the behavioral data are reported in Table 2. The best‐fitting model had a significant main effect of trial type, 𝜒^2^(3) = 6690.05, 𝑝 < 0.001 (see Supporting Information for all candidate models). Holm‐corrected post hoc tests found significant differences in mean RT between all trial types (all 𝑝 < 0.001). Figure 4 shows the full RT distributions for each trial type in this analysis.

**TABLE 2:** Descriptive statistics of the behavioral data. Mean reaction time (RT) and SD are given for all (successful) go trials, failed stops, successful stops, and (successful) ignores (SS = successful stop). Additionally, descriptive statistics for the SS and ignore trials are segmented based on the presence of partial EMG responses (PR). Failed ignore and failed go trials were excluded from the analysis due to their infrequency (participants made errors on 0.01% of go trials and 1.3% of ignore trials) and are thus not reported here.

Trial type	Proportion	Mean RT (ms)	SD
Go		498	170
Failed stop		432	132
Successful stop (SS)		641	170
SS with a partial response	39.5%	685	172
SS without a partial response	60.5%	611	167
Ignore		582	214
Ignore with a partial response	26.1%	724	213
Ignore without a partial reponse	73.9%	519	183

### 3.2 EMG Results (Including RT Results Split by Presence of Partial Responses)

Descriptive statistics summarizing CT, amplitude, and action reprogramming in stop and ignore trials are reported in Table 3. Details of best‐fitting models and all candidate models are reported in the Supporting Information.

**TABLE 3:** Descriptive statistics for cancel time (CT), the amplitude of the response‐generating RT burst, and action reprogramming for stop and ignore trials. Amplitude is relative to the mean amplitude in successful go trials for each participant, that is, values greater than 1 indicate increased amplitude. Ignore trials were significantly slower than stop trials for both CT and action reprogramming, both *p* < 0.001. The amplitude of the response‐generating RT burst was significantly lower in ignore trials without a partial response (PR) than in the other conditions (all *p* ≤ 0.036).

Measure	Trial type	Mean	SD
Cancel time (ms)	Stop	167	57
	Ignore	179	86
Amplitude of response‐generating RT burst	Stop with PR	1.08	0.27
	Stop no PR	1.06	0.25
	Ignore with PR	1.08	0.28
	Ignore no PR	1.01	0.25
Action reprogramming (ms)	Stop	230	71
	Ignore	257	109

#### 3.2.1 Effect of Partial Response on Reaction Time (RT)

There was a significant interaction between partial response and trial type (𝜒^2^[1] = 459.35, 𝑝 < 0.001), and significant main effects of both partial response (𝜒^2^[1] = 442.52, 𝑝 < 0.001) and trial type (𝜒^2^[1] = 42.68, 𝑝 < 0.001). Holm‐corrected post hoc tests revealed there were significant differences in mean RT for all possible comparisons (all 𝑝 < 0.001). In general, trials with a partial response had a longer RT than those without for both successful stop and ignore trials. Descriptive statistics are given in Table 2. See Figure 4 for RT distributions split by partial response presence.

#### 3.2.2 Cancel Time (CT)

**FIGURE 5:**
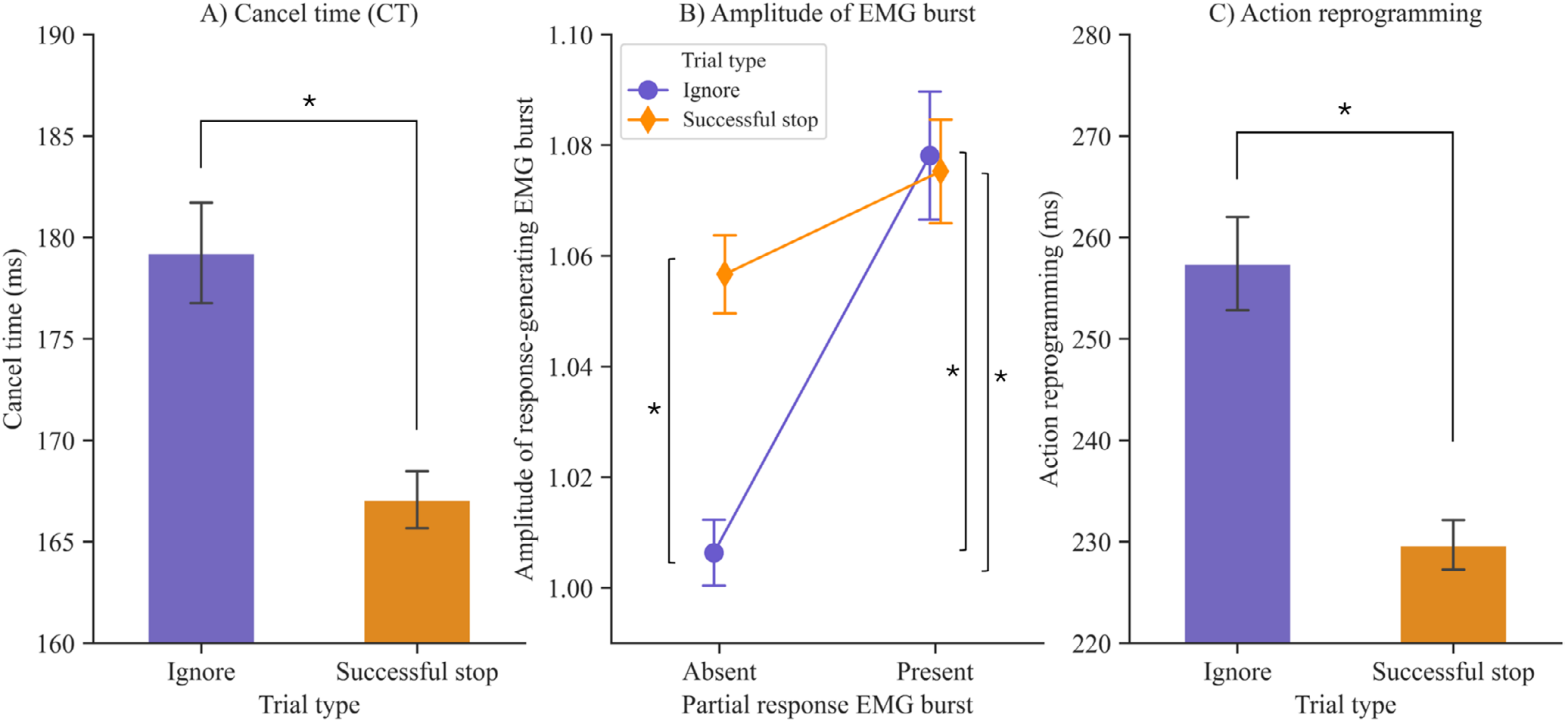
EMG results showing differences in cancel time (A), the amplitude of the response‐generating burst (B), and action reprogramming (C) in successful stop and successful ignore trials. Error bars denote SE. Significant differences (𝑝 < 0.05) are denoted with *. Cancel time is the latency of the peak of a partial response relative to the stop‐signal delay (SSD). Amplitude is relative to the mean amplitude in successful go trials. Action reprogramming is the duration between the peaks of a partial response and a response‐generating RT burst.

There was a statistically significant main effect of trial type (𝜒^2^[1] = 8.30, 𝑝 = 0.004); CT was slower in ignore trials than in successful stop trials (Table 3). The interaction effect (trial type × hand) and main effect of hand were not significant (𝜒^2^[1] = 0.001, 𝑝 = 0.975 and 𝜒^2^[1] = 0.07, 𝑝 = 0.795, respectively). See Figure 5.

#### 3.2.3 EMG Amplitude in Stop and Ignore Trials

There was a statistically significant interaction between trial type and partial response (𝜒^2^[1] = 10.49, 𝑝 = 0.001). The interaction effect is depicted in Figure 5. Holm‐corrected post hoc tests indicated that in ignore trials without a partial response, the amplitude of the subsequent response‐generating burst was significantly lower than in stop trials without a partial response (𝑝 = 0.036), ignore trials with a partial response (𝑝 < 0.001), and stop trials with a partial response (𝑝 = 0.003). There was no significant difference in the response‐generating amplitude of stop trials with and without partial responses (𝑝 = 0.403) or between ignore and stop trials with partial responses (𝑝 = 0.657). See Figure 5.

There was also a statistically significant main effect of partial response (𝜒^2^[1] = 27.57, 𝑝 < 0.001). The amplitude of the response‐generating burst was higher in trials with a partial response (𝑀 = 1.08, 𝑆𝐷 = 0.27) than trials without one (𝑀 = 1.03, 𝑆𝐷 = 0.25). The main effect of trial type was not statistically significant (𝜒^2^[1] = 1.20, 𝑝 = 0.273). See Figure 5.

#### 3.2.4 Action Reprogramming in Stop and Ignore Trials With Partial Response

There was a significant effect of trial type (𝜒^2^[1] = 12.59, 𝑝 < 0.001). The delay between partial responses and response‐generating bursts was significantly longer in ignore trials (M = 257.43 ms, SD = 109.10) than in stop trials (M = 229.69 ms, SD = 71.13). See Figure 5.

### 3.3 fNIRS Results

**FIGURE 6:**
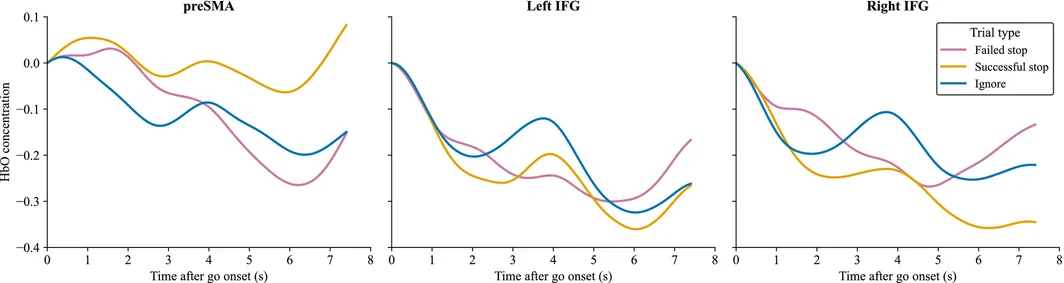
fNIRS signal for failed stop, successful stop, and ignore trials, for the preSMA, left IFG, and right IFG, averaged across participants, following go signal onset (time 0). Changes in neural activity triggered by the stimulus are reflected in Hb concentration changes, typically appearing with a 3–6 s delay. The averaged waveforms are shown here for illustrative purposes only; all statistical analyses, which focused on comparing successful stop and ignore trials, were conducted at the single‐trial level rather than on participant‐averaged time courses (see 2.5).

Averaged fNIRS signal across participants for each region for failed stops, successful stops, and ignore trials is shown in Figure 6.

#### 3.3.1 preSMA

**FIGURE 7:**
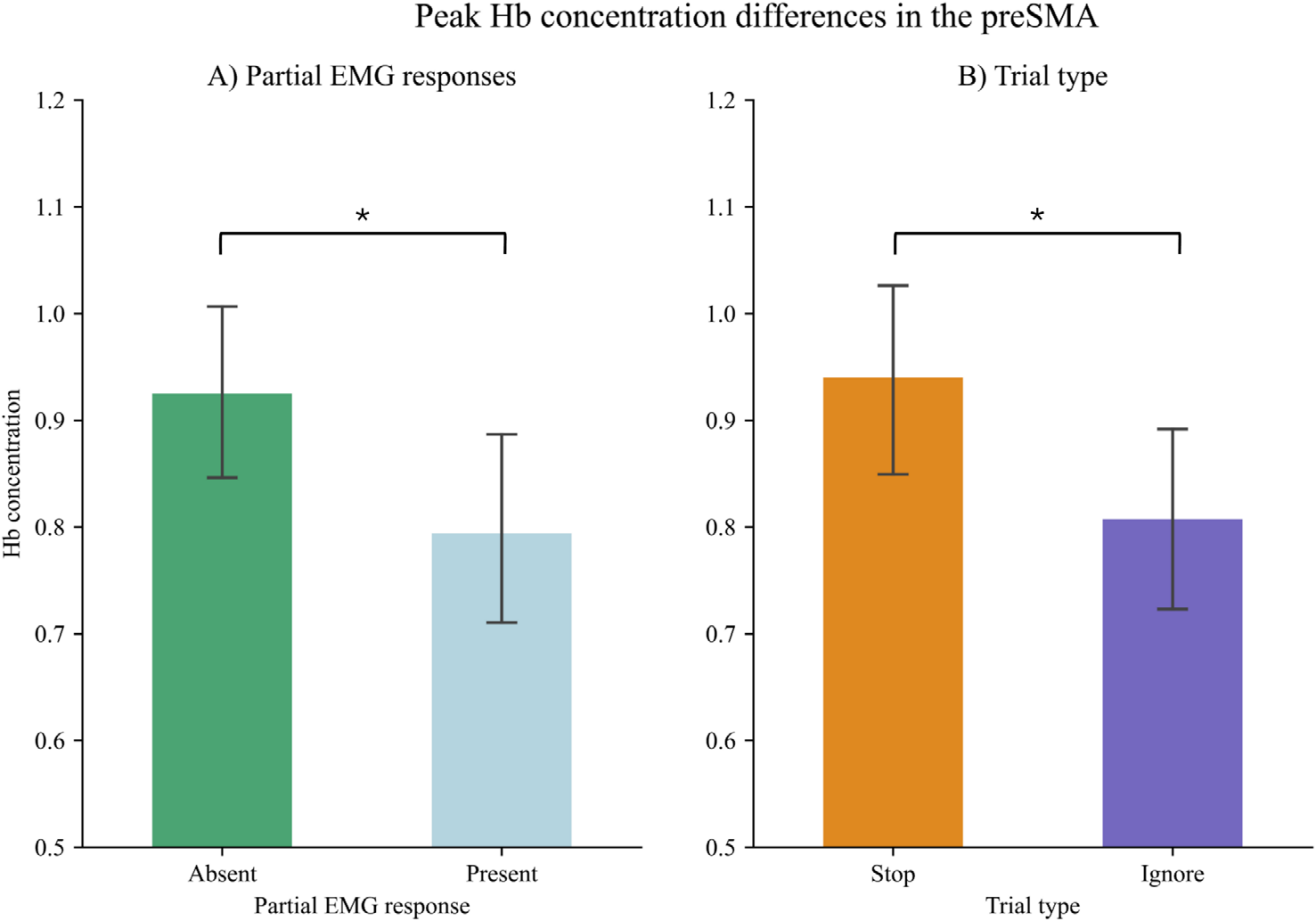
Peak oxygenated hemoglobin (Hb) concentration in the pre‐supplementary motor area (preSMA). Increased Hb concentration indicates increased neural activity in that condition. There was significantly higher peak concentration in trials without a partial EMG response (A), and significantly higher peak concentration in successful stop trials (B).

There were significant main effects of trial type and partial response (𝜒^2^[1] = 5.78, 𝑝 = 0.016 and 𝜒^2^[1] = 3.97, 𝑝 = 0.046, respectively). See Figure 7. Peak Hb concentration was significantly higher in successful stop trials (𝑀 = 0.94, 𝑆𝐷 = 1.59) than in ignore trials (𝑀 = 0.81, 𝑆𝐷 = 1.66). Peak Hb concentration was also significantly higher in trials *without* partial EMG responses (𝑀 = 0.92, 𝑆𝐷 = 1.60) than trials with them (𝑀 = 0.79, 𝑆𝐷 = 1.66). The interaction effect was not statistically significant (𝜒^2^[1] = 0.54, 𝑝 = 0.464).

#### 3.3.2 Right and Left IFG

For the RIFG, neither of the main effects nor interaction terms were statistically significant (trial type: 𝜒^2^[1] = 1.90, 𝑝 = 0.168; partial response: 𝜒^2^[1] = 0.01, 𝑝 = 0.928; interaction: 𝜒^2^[1] = 0.30, 𝑝 = 0.582). Similarly, for the LIFG, none of the main effects nor interaction terms were statistically significant (trial type: 𝜒^2^[1] = 3.12, 𝑝 = 0.077; partial response: 𝜒^2^[1] = 0.19, 𝑝 = 0.664; interaction: 𝜒^2^[1] < 0.01, 𝑝 = 0.983). The interaction effects for the RIFG and LIFG are depicted in the Supporting Information, along with all candidate models.

#### 3.3.3 Correlations

Pearson correlations were computed for each participant between mean peak Hb concentration per trial type and corresponding behavioral and EMG indices (go RT, ignore RT, failed stop RT, and CT) for each ROI (12 correlations in total). Following FDR correction, no correlations were statistically significant (all *p* > 0.05). Full results are reported in the Supporting Information.

## 4 Discussion

Action cancellation has most commonly been studied using the SST, which has provided a foundational understanding of inhibitory control. Behavior in the standard SST likely reflects multiple processes, which are often investigated using variants of the SST. In the present study, we used a response‐and stimulus‐selective SST, which allowed for the isolation of attentional orienting by means of ignore cues and required the cancellation of only part of the ongoing movement, allowing the investigation of adaptive movement, rather than outright cancellation. By combining behavioral, EMG, and fNIRS measures, we examined how action cancellation and motor adaptation unfold at the level of the motor system, and how the canonical cortical stopping regions, the preSMA and IFG, may differentially support these operations. In addition, prior work has identified partial EMG responses during successful stopping, reflecting instances in which motor activity is initiated and then terminated, without producing an overt response. Although these partial responses are typically interpreted as markers of late‐stage inhibition, their relationship to the neural systems underlying action cancellation remains unclear. Accordingly, we also investigated the neural correlates of partial EMG responses and their behavioral consequences within this framework.

### 4.1 CT and Action Reprogramming Are Slower in Ignores Than in Stop Trials

We assessed differences in cancel time (CT; the latency of the partial EMG response) and action reprogramming (the delay between the partial EMG response and subsequent response‐generating EMG burst, which indexes the time taken to “reprogram” an action) between stop and ignore trials. Action reprogramming was slower for ignore trials than stop trials by approximately 30 ms (Table 3), consistent with previous findings (Weber, Salomoni, and Hinder 2024). CT was longer in ignore trials compared to stop trials (Table 3).

It has been suggested that the neurophysiological signatures associated with the cancel/retune process may scale with the degree of “retuning” required (Hervault and Wessel 2025). More extensive modifications (e.g., changing multiple components of a movement) appear to have greater neurophysiological input than those with more minimal adjustments, evident in some EEG signatures (specifically the P300: see Hervault and Wessel 2025). One may, therefore, expect the delay between partial responses and response‐generating EMG bursts to be shorter in ignore trials than in stop trials, given ignore trials just require the continuation of the planned movement, while stop trials require either modification of this movement or cancellation and execution of a new movement. These findings are inconsistent with this prediction, adding to current challenges reconciling other neurophysiological signatures with EMG measures (for discussion, see Kemp and Weber 2026), and representing an important direction for future research.

### 4.2 IFG and preSMA Activity in Stop Trials Compared to Ignore Trials

To isolate the neural correlates of stopping vs. attentional capture, we examined neural activity in the preSMA, RIFG, and LIFG in (successful) stop and ignore trials. We found increased neural activity in the successful stops compared to ignore trials in the preSMA, but not in the left or right IFG (Figure 7 and Supporting Information), consistent with fMRI evidence (Sharp et al. 2010). This also supports the PTC framework, which suggests that the IFG triggers the pause process in response to any infrequent or unexpected stimulus and should, thus, be active in both stop and ignore conditions (Diesburg and Wessel 2021). Furthermore, the preSMA is postulated to initiate the cancel/retune process (Diesburg and Wessel 2021; Tatz et al. 2021), meaning preSMA activity would be expected in stop but not ignore trials, as was observed here. Event‐related potential (ERP) studies have identified two stop‐specific neural signatures: a right‐frontally distributed stop N2, potentially the “pause” process, and a subsequent stop‐specific P3, proposed as an index of action cancellation itself (Hervault et al. 2024; Hervault and Wessel 2025; Kenemans et al. 2023), providing further evidence of dissociable neural mechanisms for these processes.

IFG activation in response to unexpected stimuli has been observed across a variety of paradigms (Boehler et al. 2011; Chatham et al. 2012; Erika‐Florence et al. 2014; Sebastian et al. 2021), consistent with the interpretation that its function is domain‐general and not limited to action cancellation (see also Wessel and Anderson 2024). However, we note that evidence from electrocorticography (ECoG), which offers extremely high spatio‐temporal resolution, suggests that during stop preparation, preSMA activity may *precede* the IFG (Swann et al. 2012), challenging the idea that these regions instantiate sequential processes (pause, then cancel). Additionally, some accounts propose that the initial pause mechanism may be sufficient to realize action cancellation, or that the “pause” and “cancel” mechanisms may actually comprise one unified process (Chaudhuri et al. 2025; Hannah et al. 2022). However, as mentioned above, ERP findings appear to support separable neural mechanisms underpinning action cancellation, which are yet to be reconciled with accounts of a single, unified process. Taken together, there is a clear need for additional research to clarify the functional contributions of these regions in action cancellation; future work may consider the combination of temporally‐ and spatially‐sensitive methods to best elucidate the timing and origins of these signatures.

### 4.3 Neural Activity and Partial EMG Responses

Partial EMG responses are increasingly widely used as an index of action cancellation, as they provide an objective and temporally precise measure of an action being initiated and interrupted (Raud et al. 2022; Salomoni et al. 2025). It has recently been proposed that successful stop trials with partial responses represent true instances of action cancellation, whereas those without partial responses instead reflect action withholding (Wadsley and Greenhouse 2024). Action withholding occurs when participants inhibit their movement *before* they have begun to respond, rather than initiating and then cancelling the response (Schachar et al. 2007). Thus, we compared neural activity between trials with and without partial responses, finding increased preSMA activity in trials *without* partial responses, but no significant differences in IFG activity (Figure 7).

Action execution and stopping are likely realized via three cortico‐basal ganglia pathways, with the direct pathways supporting movement execution (Graybiel 2000; Rocha et al. 2023) and action cancellation likely by the hyperdirect and/or indirect pathways (Aron 2007; Bingham et al. 2023; Coudé et al. 2018; Schroll and Hamker 2013). These pathways initiate in the cortex and transmit to basal ganglia regions, the thalamus, and primary motor cortex (M1).^1^ Action cancellation signals can likely interrupt activity at multiple points along the direct pathway, for example, by increasing thalamic inhibitory drive to M1 or inhibiting input to the striatum via the globus pallidus (Schmidt and Berke 2017). In such cases, neural activity would be interrupted well before the go command is issued from M1, preventing transmission to the effector muscles and precluding a detectable EMG response. Thus, action cancellation mechanisms can feasibly underlie successful stopping both with and without partial responses, although distinguishing between action withholding and action cancellation in trials without partial EMG activity remains a challenge.

The observed increase in preSMA activity in trials without partial responses may reflect the engagement of proactive inhibition mechanisms. Proactive inhibition is a strategic, preparatory mechanism that operates to increase the likelihood of successful stopping through pre‐emptive modulation of corticospinal excitability and delaying of action initiation (Bundt and Huster 2024; Kemp et al. 2026; van den Wildenberg et al. 2022). In contrast, reactive inhibition occurs where participants do not prepare for the upcoming stop, and the cessation of the motor process begins entirely after stop signal onset. It has been proposed that both reactive and proactive inhibition are required for effective inhibitory control (Criaud and Boulinguez 2013) and critically, the preSMA has been associated with both reactive and proactive inhibition (Criaud and Boulinguez 2013; Kenemans 2015; Meyer and Bucci 2016; Obeso et al. 2013), with recent evidence suggesting it may even be the driver of these two processes (Zhu et al. 2024). Evidence from non‐human primates suggests that different neural populations within the preSMA may modulate these two types of inhibition (Chen et al. 2010). The observed increase of preSMA activity in trials without partial responses may reflect both proactive *and* reactive inhibitory processes at work, whereas trials with partial responses may represent instances of reactive inhibition alone. Proactive inhibitory processes can also markedly reduce corticomotor excitability (Kemp et al. 2026), precluding an observable EMG response.

### 4.4 Limitations

In the current study, we assessed the ROIs individually, running models on their changes in hemoglobin concentration and assessing whether they differed between trial conditions or in instances with and without partial responses. Given that these regions are different sizes with differing vasculature, and that the preSMA is further from the skull than the IFG, we chose to assess these regions individually (in separate models) due to fundamental differences in their baseline activity. We maintain that direct comparisons here are limited in meaning. Nonetheless, this means that we cannot make statements about relative amounts of Hb concentration (e.g., we cannot state if the IFG is *more* associated with ignore signals than the preSMA: Nieuwenhuis et al. 2011). Relatedly, we note that we defined the ROIs according to the MNI template (see 2.3.2), which does not capture individual variability in neuroanatomy and could result in imperfect alignment between the predefined ROIs and the underlying cortical structures.

Regarding the task, we utilized a response‐ and stimulus‐selective stopping paradigm, in order to focus on disentangling attentional capture and action reinitiation. Nonetheless, we acknowledge that there are many variations of the SST, including the standard paradigm (Verbruggen et al. 2019), which can be used to examine other aspects of action cancellation (Weber et al. 2026). Future work may utilize these to more fully elucidate the neural and physiological mechanisms underpinning action cancellation. Additionally, a limit of the behavioral analysis is that the response times for the left and right hand were averaged. While this was motivated by the tight temporal coupling generally observed between effectors, it necessarily obscures hand‐specific variability. Future work may combine and contrast variations of the SST to more fully elucidate the neural and physiological mechanisms underpinning action cancellation.

Finally, we note that fNIRS is a technique with poor temporal resolution, meaning that its signatures cannot be used to determine the precise timing of neural activation relative to behavioral events. Future work may consider combining fNIRS with more temporally precise techniques such as EEG to better characterize the spatio‐temporal dynamics of action cancellation.

## 5 Conclusion

We assessed the role of the preSMA and IFG in action cancellation, attentional orienting, and partial EMG responses using a novel fNIRS paradigm. We found that the preSMA was more uniquely involved in action cancellation while the IFG appeared equally involved in action cancellation and attentional capture. There was increased preSMA activity in trials without partial EMG responses; we propose that this may result from increased proactive inhibition in these trials.

## Author Contributions

**Sarah A. Kemp:** conceptualization, data curation, formal analysis, investigation, methodology, project administration, software, visualization, validation, writing – original draft, writing – review and editing. **Pierre‐Louis Bazin:** methodology, software, supervision, validation, writing – review and editing. **Rebecca J. St George:** data curation, funding acquisition, resources, supervision, validation, writing – review and editing. **Mark R. Hinder:** conceptualization, funding acquisition, resources, supervision, writing – review and editing. **Sauro Salomoni:** data curation, methodology, software, validation, writing – review and editing. **Luke Pash:** data curation, investigation, writing – original draft, writing – review and editing.

## Funding

This work was supported by the Australian Research Council Discovery program (DP200101696) and a University of Tasmania Graduate Research Scholarship.

## Conflicts of Interest

The authors declare no conflicts of interest.

## Supporting information


**Figure S1:** Differences in neural activity in the preSMA, left IFG, and right IFG in stop and ignore trials. Error bars denote SE. Generalized linear mixed models found no di erences between trials with and without partial EMG responses in any of the regions of interest. The preSMA had significantly higher activation in the stop trials than in ignore. The right and left IFG did not show any di erences in neural activity between stop and ignore trials.

## Data Availability

The data that support the findings of this study are available on request from the corresponding author. The data are not publicly available due to privacy or ethical restrictions.

## Endnotes

^1^
In humans, the pathways are typically characterized as follows: the direct pathway projects from the cortex to the striatum, globus pallidus (GP) interna, thalamus, and M1; the indirect pathway from cortex to striatum, GP externa, STN, GP interna, thalamus, and M1; the hyperdirect pathway from cortex to STN, substantia nigra, GP interna, thalamus, and M1 (Bingham et al. 2023; W. Chen et al. 2020; Graybiel 2000; Nambu et al. 2002; Polyakova et al. 2020; Rocha et al. 2023).
