# Supplementary material for: Cortical Contributions to Attentional Orienting and Response Cancellation in Action Stopping
Source: Psychophysiology. 2026 Sep 7;63(9):e70397. doi: 10.1111/psyp.70397 (PMC13548964; doi:10.1111/psyp.70397)
Supplement: Supplementary file 1 [file PSYP-63-e70397-s001.pdf]

Cortical contributions to attentional orienting and partial response EMG in the stop-signal task

Supplementary Materials

3.1 Behavioural results

Model candidates from 3.1, which assessed RT differences between different trial types (go, successful stops, failed stops, and ignores). The best-fitting model is emphasised in **bold**.

Candidate	*Distribution & link function*	*Random effects structure*	*BIC*
Model 1	Gaussian + loglink	1 | Participant	253854
Model 2	Gaussian + loglink	1 + Trial | Participant	254352
Model 3	Gamma + identity	1 | Participant	248656
**Model 4**	**Gamma + identity**	**1 + Trial | Participant**	**248162**

3.2 EMG results

3.2.1 Effect of partial response on reaction time

Model candidates from 3.2.1, which the effect of partial response on reaction time in stop and ignore trials. Fixed effects were trial type (stop, ignore) and partial response EMG (present, absent). Random effects were trial type (TT), partial response (PR), or their interaction. Best-fitting model is emphasised in **bold**.

Candidate	*Distribution & link function*	*Random effects structure*	*BIC*
Model 1	Gaussian + loglink	1 | Participant	55966
Model 2	Gaussian + loglink	1 + TT | Participant	56233
Model 3	Gaussian + loglink	1 + PR | Participant	55982
Model 4	Gaussian + loglink	1 + PR + TT | Participant	56412
Model 5	Gaussian + loglink	1 + PR + TT + PR*TT | Participant	56833
Model 6	Gamma + identity	1 | Participant	55368
Model 7	Gamma + identity	1 + TT | Participant	55318
Model 8	Gamma + identity	1 + PR | Participant	55333
Model 9	Gamma + identity	1 + PR + TT | Participant	55306
**Model 10**	**Gamma + identity**	**1 + PR + TT + PR*TT | Participant**	**55303**

3.2.2 Cancel time in stop and ignore trials

Model candidates from 3.2.2, which examined cancel time in stop and ignore trials. Fixed effects were trial type (stop, ignore) and hand (cued, uncued). Random effects could be trial type (TT), hand (H), or their interaction. Best-fitting model is emphasised in **bold**.

Candidate	*Distribution & link function*	*Random effects structure*	*BIC*
Model 1	Gaussian + loglink	1 | Participant	32597
Model 2	Gaussian + loglink	1 + TT | Participant	32625
Model 3	Gaussian + loglink	1 + H | Participant	32628
Model 4	Gaussian + loglink	1 + H + TT | Participant	33102
Model 5	Gaussian + loglink	1 + H + TT + H*TT | Participant	33439
Model 6	Gamma + identity	1 | Participant	31568
**Model 7**	**Gamma + identity**	**1 + TT | Participant**	**31562**
Model 8	Gamma + identity	1 + H | Participant	31571
Model 9	Gamma + identity	1 + H + TT | Participant	31574
Model 10	Gamma + identity	1 + H + TT + H*TT | Participant	31590

3.2.3 Effect of partial response on response-generating EMG amplitude

Model candidates from 3.2.3, wherein we examined the effect of a partial response on the ultimate amplitude of the response-generating burst in stop and ignore trials. Fixed effects were trial type (stop, ignore) and partial response (present, absent). Random effects could be trial type (TT), partial response (PR), or their interaction. Best-fitting model is emphasised in **bold**.

Candidate	*Distribution & link function*	*Random effects structure*	*BIC*
Model 1	Gaussian + loglink	1 | Participant	541
**Model 2**	**Gaussian + loglink**	**1 + TT | Participant**	**511**
Model 3	Gaussian + loglink	1 + PR | Participant	548
Model 4	Gaussian + loglink	1 + PR + TT | Participant	531
Model 5	Gaussian + loglink	1 + PR + TT + PR*TT | Participant	550
Model 6	Gamma + identity	1 | Participant	941
Model 7	Gamma + identity	1 + TT | Participant	913
Model 8	Gamma + identity	1 + PR | Participant	948
Model 9	Gamma + identity	1 + PR + TT | Participant	935
Model 10	Gamma + identity	1 + PR + TT + PR*TT | Participant	960

3.3 fNIRS results

Model candidates from 3.3, where we examined differences in HbO concentration in the preSMA, RIFG, and LIFG in stop and ignore trials with and without partial EMG response. Fixed effects were trial type (stop, ignore) and partial response (present, absent). Random effects could be trial type (TT), partial response (PR), or their interaction. Best-fitting models are emphasised in **bold**.

3.3.1 preSMA

Candidate	*Distribution & link function*	*Random effects structure*	*BIC*
**Model 1**	**Gaussian + identity (LMM)**	**1 | Participant**	**10385**
Model 2	Gaussian + identity (LMM)	1 + TT | Participant	10399
Model 3	Gaussian + identity (LMM)	1 + PR | Participant	10401
Model 4	Gaussian + identity (LMM)	1 + TT + PR | Participant	10422
Model 5	Gaussian + identity (LMM)	1 + TT + PR + PR*TT | Participant	10453

3.3.2 Right and left IFG

Right IFG

Candidate	*Distribution & link function*	*Random effects structure*	*BIC*
**Model 1**	**Gaussian + identity (LMM)**	**1 | Participant**	**11388**
Model 2	Gaussian + identity (LMM)	1 + TT | Participant	11401
Model 3	Gaussian + identity (LMM)	1 + PR | Participant	11428
Model 4	Gaussian + identity (LMM)	1 + TT + PR | Participant	11424
Model 5	Gaussian + identity (LMM)	1 + TT + PR + PR*TT | Participant	11456

Left IFG

Candidate	*Distribution & link function*	*Random effects structure*	*BIC*
**Model 1**	**Gaussian + identity (LMM)**	**1 | Participant**	**12010**
Model 2	Gaussian + identity (LMM)	1 + TT | Participant	12019
Model 3	Gaussian + identity (LMM)	1 + PR | Participant	12026
Model 4	Gaussian + identity (LMM)	1 + TT + PR | Participant	12043
Model 5	Gaussian + identity (LMM)	1 + TT + PR + PR*TT | Participant	12089

3.3 fNIRS results

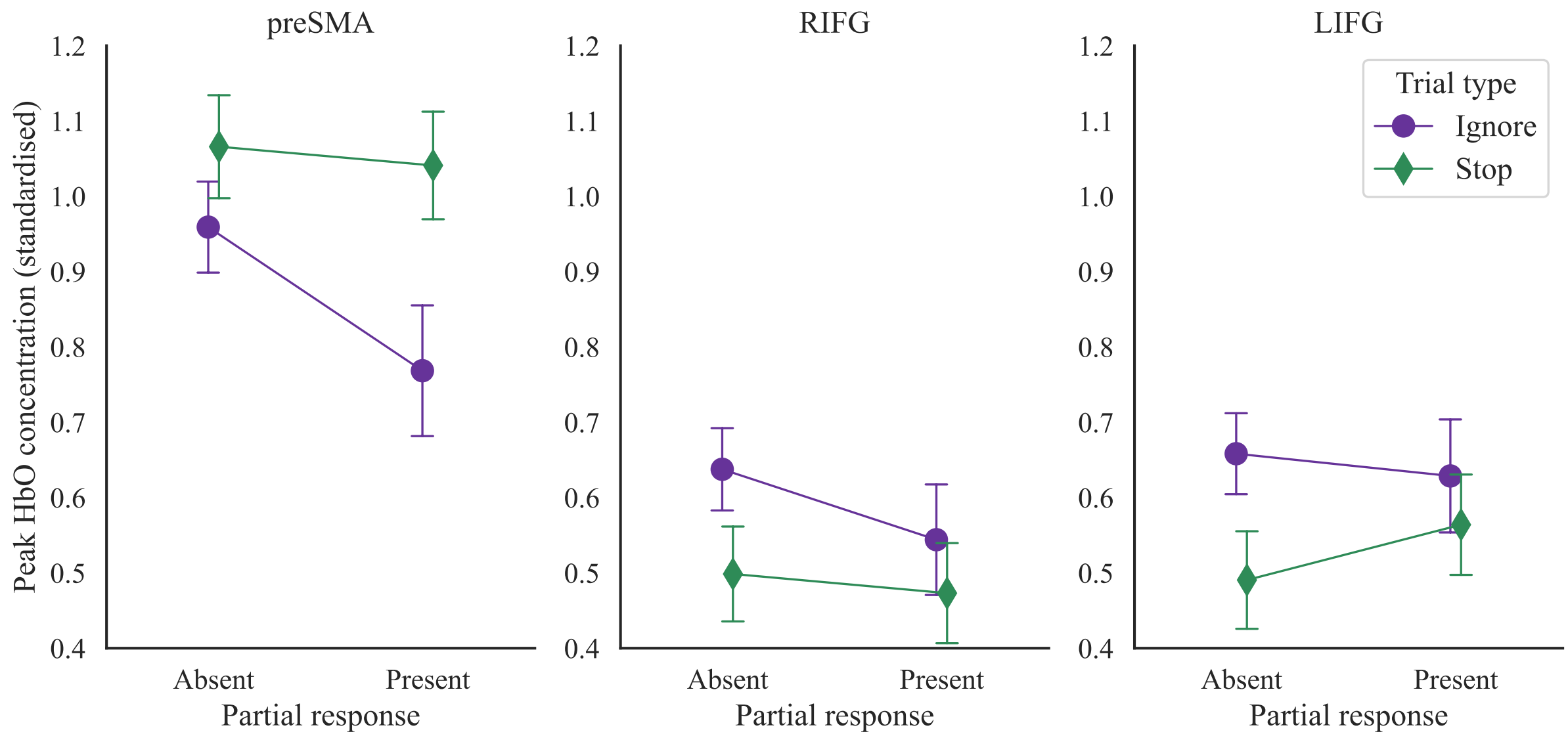

Figure S1. Differences in neural activity in the preSMA, left IFG, and right IFG in stop and ignore trials. Error bars denote SE. Generalised linear mixed models found no differences between trials with and without partial EMG responses in any of the regions of interest. The preSMA had significantly higher activation in the stop trials than in ignore. The right and left IFG did not show any differences in neural activity between stop and ignore trials

3.3.3 Correlations

fNIRS/mean peak Hb concentration	*Behavioural/EMG variable*	*Region*	*Pearson* r *(95% CI)*	***p-value***	
				Original	*FDR corrected*
Go trials	Mean go RT	preSMA	-0.18 (-0.52, 0.21)	0.356	0.972
		RIFG	-0.03 (-0.40, 0.35)	0.874	0.972
		LIFG	-0.01 (-0.38, 0.37)	0.969	0.972
Ignore trials	Mean ignore RT	preSMA	-0.11 (-0.47, 0.27)	0.572	0.972
		RIFG	0.13 (-0.26, 0.48)	0.527	0.972
		LIFG	0.12 (-0.27, 0.47)	0.542	0.972
Successful stop trials	Mean cancel time (EMG)	preSMA	-0.01 (-0.38, 0.37)	0.972	0.972
		RIFG	0.50 (0.16, 0.74)	0.007	0.080
		LIFG	0.12 (-0.27, 0.47)	0.542	0.972
Failed stop trials	Mean failed stop RT	preSMA	0.19 (-0.19, 0.53)	0.324	0.972
		RIFG	0.05 (-0.33, 0.42)	0.783	0.972
		LIFG	0.06 (-0.32, 0.43)	0.750	0.972

Results of all correlations, which assessed associations between mean peak Hb concentration (fNIRS) for each trial type and the relevant behavioural/EMG index.
